# Detection of diabetes from whole-body MRI using deep learning

**DOI:** 10.1172/jci.insight.146999

**Published:** 2021-11-08

**Authors:** Benedikt Dietz, Jürgen Machann, Vaibhav Agrawal, Martin Heni, Patrick Schwab, Julia Dienes, Steffen Reichert, Andreas L. Birkenfeld, Hans-Ulrich Häring, Fritz Schick, Norbert Stefan, Andreas Fritsche, Hubert Preissl, Bernhard Schölkopf, Stefan Bauer, Robert Wagner

**Affiliations:** 1 Department of Computer Science, ETH Zürich, Zürich, Switzerland.; 2 Department of Radiology, Section on Experimental Radiology, Eberhard-Karls University Tübingen, Tübingen, Germany.; 3 Institute for Diabetes Research and Metabolic Diseases, Helmholtz Center Munich, University of Tübingen, Tübingen, Germany.; 4 German Center for Diabetes Research, Neuherberg, Germany.; 5 Werner Siemens Imaging Center, Tübingen, Germany.; 6 Max Planck Institute for Intelligent Systems, Department of Empirical Inference, Tübingen, Germany.; 7 Department for Diagnostic Laboratory Medicine, Institute for Clinical Chemistry and Pathobiochemistry, University Hospital Tübingen, Tübingen, Germany.; 8 Department of Internal Medicine, Division of Diabetology, Endocrinology and Nephrology, Eberhard-Karls University Tübingen, Tübingen, Germany.; 9 Institute of Robotics and Intelligent Systems, ETH Zürich, Zürich, Switzerland.; 10 Department of Gynecology and Obstetrics, University Hospital Tübingen, Tübingen, Germany.; 11 Department of Intelligent Systems, KTH Stockholm, Stockholm, Sweden.

**Keywords:** Endocrinology, Metabolism, Diabetes, Diagnostic imaging, Obesity

## Abstract

Obesity is one of the main drivers of type 2 diabetes, but it is not uniformly associated with the disease. The location of fat accumulation is critical for metabolic health. Specific patterns of body fat distribution, such as visceral fat, are closely related to insulin resistance. There might be further, hitherto unknown, features of body fat distribution that could additionally contribute to the disease. We used machine learning with dense convolutional neural networks to detect diabetes-related variables from 2371 T1-weighted whole-body MRI data sets. MRI was performed in participants undergoing metabolic screening with oral glucose tolerance tests. Models were trained for sex, age, BMI, insulin sensitivity, HbA1c, and prediabetes or incident diabetes. The results were compared with those of conventional models. The area under the receiver operating characteristic curve was 87% for the type 2 diabetes discrimination and 68% for prediabetes, both superior to conventional models. Mean absolute regression errors were comparable to those of conventional models. Heatmaps showed that lower visceral abdominal regions were critical in diabetes classification. Subphenotyping revealed a group with high future diabetes and microalbuminuria risk.Our results show that diabetes is detectable from whole-body MRI without additional data. Our technique of heatmap visualization identifies plausible anatomical regions and highlights the leading role of fat accumulation in the lower abdomen in diabetes pathogenesis.

## Graphical abstract

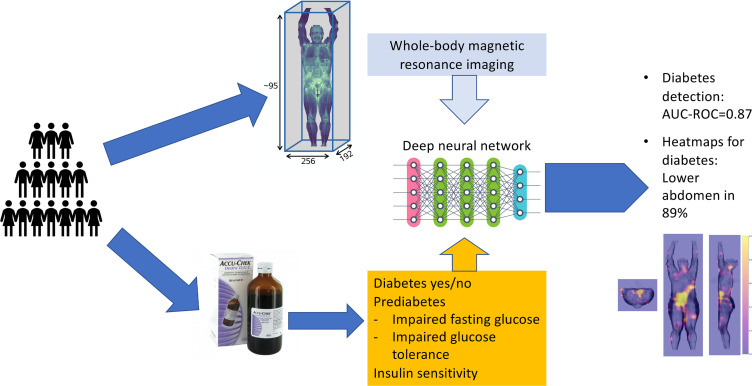

## Introduction

Currently around 500 million individuals worldwide have diabetes, which has a dramatically rising prevalence (1). Most diabetes cases are type 2 diabetes, which is a condition determined by a combination of reduced insulin action in the insulin target tissues, i.e., insulin resistance, and an insufficient compensation for this insulin resistance due to an impaired insulin secretion. Epidemiologically obesity is the main driver of insulin resistance (2), but excess body fat mass is neither a prerequisite nor a guarantee for insulin resistance. There are individuals who remain metabolically healthy despite being obese (3), while others develop insulin resistance despite normal body weight (4). This is because the distribution of fat within the human body crucially determines its metabolic role. Individuals with mostly deep abdominal and visceral fat accumulation are more prone to develop insulin resistance compared with individuals with mostly subcutaneous fat deposition (5). On the other hand, subcutaneous abdominal and thigh fat seem to act as protective triglyceride dumps in the body, which preserve insulin sensitivity by confining fat to metabolically inert body regions (6, 7). Specific fat compartments, such as fat depots near arteries, seem to play distinctive roles in the pathophysiology of insulin resistance, insulin secretion, and probably also the manifestation of metabolic complications (8). Some of these perivascular fat depots, such as those near the brachial artery, have been shown to associate with insulin resistance (9). Fat tissue in the renal sinus could contribute to nephropathy (10, 11). Furthermore, pancreatic fat deposition associates with reduced insulin secretion and may be involved in the decompensation of insulin secretion and thus in the pathogenesis of diabetes (12–14). However, it is challenging to assess the aggregate effect of fat distribution on diabetes. Simple anthropometric variables of fat distribution, such as waist and hip circumference, are not very accurate and provide only limited information on the distribution of fat over the body. A more accurate measurement of fat distribution can be achieved by whole-body T1-weighted MRI (15), which by design contrasts fat and water signals as a distribution of gray scale voxels over the body. It is possible to perform a segmentation of MR images to quantify specific predetermined regions, but this approach is laborious and could be biased by predefined areas of interest. We therefore investigated if 3-dimensional whole-body MR tomograms could be applied in an unbiased way to determine if the represented individual had diabetes at the time of the scan. Adequately trained machine-learning models have recently been very successful in associating high-dimensional data with medical labels (16). Although it is notoriously challenging to derive human-readable information on key patterns of machine-learning classifiers (17), we also aimed to extract information on the anatomical regions decisive in establishing these associations.

## Results

### Model training for diabetes and related labels.

Sex classification converged to approximately 99% area under the receiver operating characteristic curve (AUROC) within the first approximately 25 epochs on the training set. All other labels tended to take considerably longer to converge, and individual performances varied with different network parameters. While sex classification seemed to be easily feasible for the network, the smoothed AUROC scores for the diabetes labels mostly peaked at approximately 85% for diabetes and approximately 70% for prediabetes and the extended diabetes label. As for the regression tasks, all of the predictive performances slightly varied. However, with different network parameters they generally converged to approximately 5%–15% mean absolute error (MAE) on the normalized training labels before starting to overfit. In all models, the lowest MAE has been reached for the estimation of BMI. The results of classifications and regressions achieved by the potentially novel dense convolutional neural networks in the optimal model are shown in Table 1.

**Figure 1:**
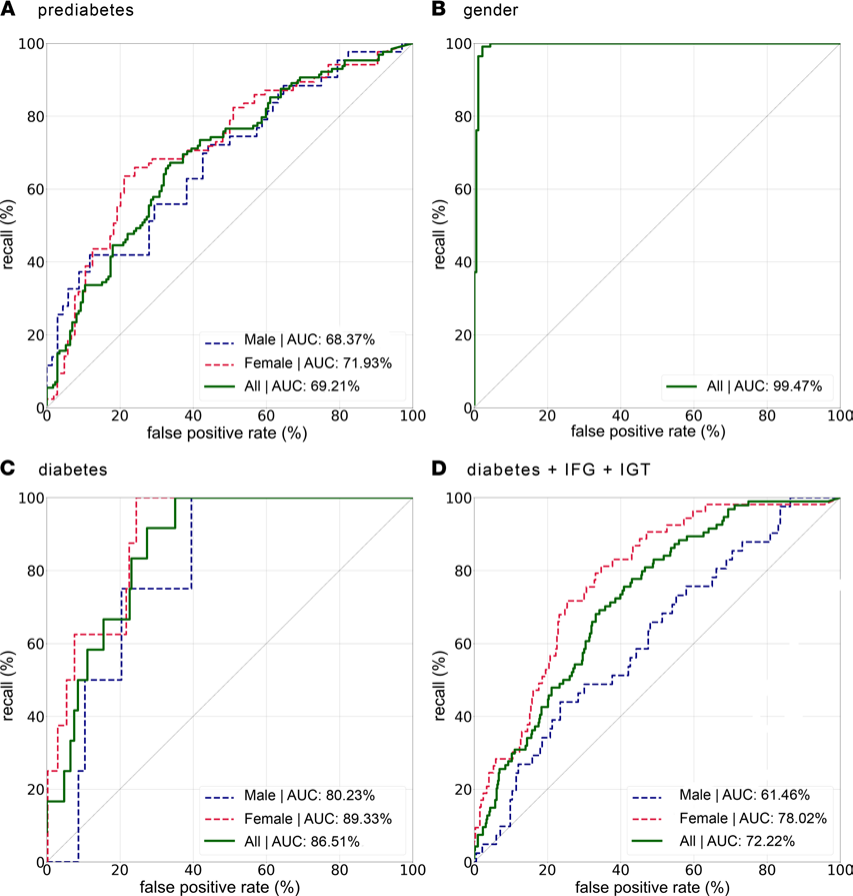
Diagnostic accuracy of the machine-learning classifiers. Receiver operating characteristic curves for the detection of prediabetes (**A**), sex (**B**), diabetes (**C**), and diabetes with impaired fasting glucose and impaired glucose tolerance (diabetes+ IFG + IGT, **D**) by the dense convolutional neural network.

The AUROC for classification of diabetes was 0.87. Receiver operator characteristics curves with and without stratification for sex are shown in Figure 1. As a comparison for the dense convolutional neural networks, conventional models were trained using body fat volumes determined by fat compartment segmentation.

A normalized MAE of 0.17 for age is equivalent to ±10 years average error. Similarly, a normalized BMI MAE of 0.07 represents an average error of ± 2kg/m2, and 0.13 normalized HbA1c MAE equals ±0.4%. Finally, the insulin sensitivity error of 0.26 corresponds to an average error of ±10.2 AU, which represents the weakest regression performance among the continuous outcome variables.

### Sensitivity analyses with different model setups.

We also tested the diagnostic precision of different model setups for the labels sex, prediabetes, and diabetes and the diabetes label extended by impaired fasting glucose and impaired glucose tolerance (IFG+IGT) (Supplemental Table 2; supplemental material available online with this article; https://doi.org/10.1172/jci.insight.146999DS1). Part of these alternative models used images cropped to torso only or abdomen only (Supplemental Figure 3). As additional augmentation technique, we tested random zooming on the images. Detection of sex was not affected by the restricted images, but the diabetes and prediabetes labels reached lower AUROC values. Interestingly, diabetes detection was only slightly affected when using abdominal images, and these techniques had no relevant effect on the detection of diabetes with IFG+IGT. With model training rerun using only the first scan from each participant, we yielded lower AUROC for diabetes, but the prediabetes and the extended diabetes labels showed comparable diagnostic precision to the original data set.

### Target-specific gradient maps.

**Figure 2:**
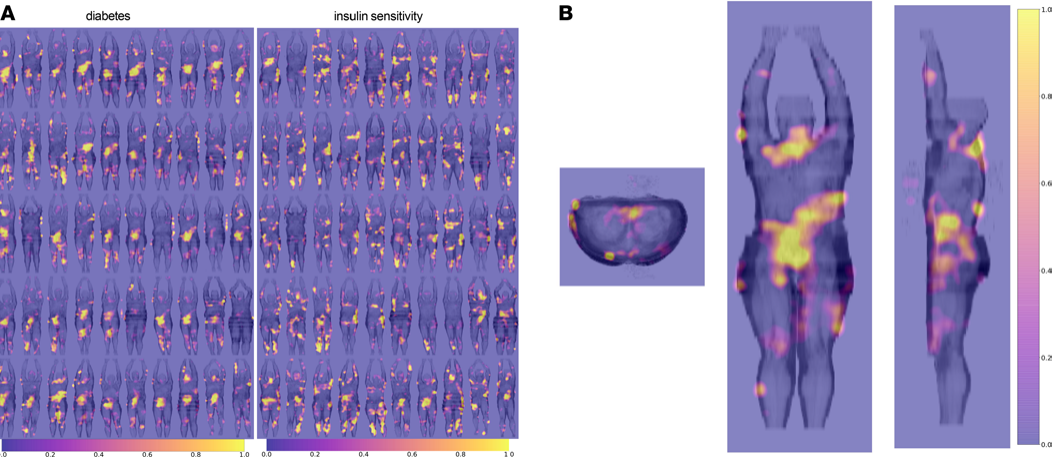
Gradient maps visualizing voxels with large influence on the classification/regression outcome. (**A**) Gradient maps for diabetes and insulin sensitivity, computed for 50, randomly selected, persons with prediabetes. The body scans, as well as the gradient maps, were averaged along the coronal projection to generate 2-dimensional representations. (**B**) An example gradient heatmap for the diabetes label in 3 projections. For assignment of gradient maps to body regions by raters, similar 3-dimensional gradient map representations, were used.

We computed attention heatmaps to acquire information about the reasoning behind predictions and to provide visualizations for further analyses. Comparison plots of heatmaps for 50 randomly selected samples for the detection of diabetes and insulin sensitivity are shown in Figure 2A The highlighted areas were assigned to prespecified anatomic regions by 3 clinicians who had expertise in the interpretation of medical imaging. For each of the 8 traits, the human experts rated 100 images presented in 3 dimensions (see example in Figure 2B). Interrater agreement was 76%.

Mean percentages for the predefined anatomical regions appearing in the heatmaps are shown in Table 2. The deep lower abdominal (visceral) region was associated with most cases of diabetes classification (89%). This region also seemed to be important, however, less prominent, for classifying diabetes with IFG+IGT cases (84%) and prediabetes cases (69%). For the classification of sex, the upper thorax region, including the breasts, played the major role (73% highlighted). Arms and upper legs were also important (67 and 61%, respectively). The upper leg region was also often highlighted in the heatmaps of the regression on BMI (64%) and insulin sensitivity (70%).

### Clusters based on the embedding layer of MRI scans.

**Figure 3:**
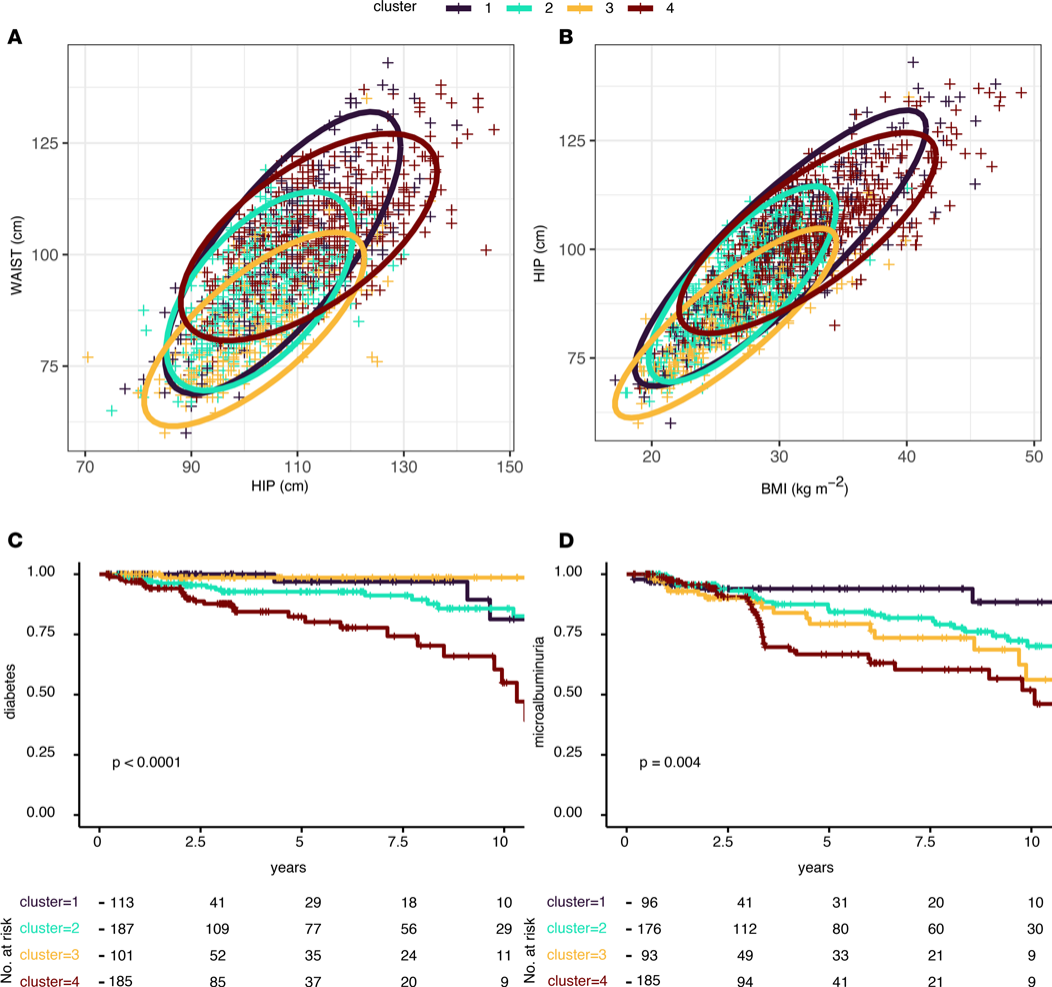
Partitioning of MRI images. Data-driven clustering was performed from embedding layers, which are numeric representations of MRI scans generated during inference (*n* = 2048). The MRI-based clusters have different distributions of waist and hip circumference (**A**) and BMI (**B**). For the participants with follow-up data, these MRI-data based clusters also define different risk profiles not only for new-onset diabetes (*n* = 586) (**C**), but also for the diabetes complication microalbuminuria (*n* = 550) (**D**). Diagrams showing incidence-free survival were compared with log-rank tests.

Of the highly complex MRI scans, the training process generated vectors with 128 values per image. These are called embeddings and contain all relevant information from the scans. To investigate whether this information contained in whole-body MRI scans can be used for the prediction of metabolic features, we performed sex-stratified data-driven clustering on the embeddings. The clusters solely based on data from the embeddings delineated groups with different anthropometric and glycemic features (Figure 3, A and B, and Table 3). Furthermore, they also predicted future diabetes (Figure 3C, n = 586 with follow-up data, mean follow-up 4 ± 3.7 years, number of events = 48, P < 0.0001) and the development of microalbuminuria (Figure 3D, n = 550 with follow-up data, mean follow-up 4.3 ± 3.6 years, number of events = 95, P = 0.004). Anthropometric variables were different across clusters, but the association of cluster 4 with increased risk of diabetes and microalbuminuria was still significant after adjustment for sex, age and BMI (P = 0.01 and P = 0.03, respectively). In addition, the association of cluster 4 with these outcomes was not explained by differences in baseline glycemia (P = 0.02 for future diabetes after adjusting for baseline glycated hemoglobin) or baseline urinary albumin-to-creatinine ratio (uACR) (P = 0.04 for future microalbuminuria after adjusting for baseline uACR, n = 441, events = 76).

## Discussion

Here, we tested if presence of diabetes can be identified from specific patterns of body fat distribution assessed by MRI. With a machine-learning approach on more than 2000 whole-body MRI data sets, we produced excellent classification results that were superior to those from state-of-the-art statistical modeling of body fat compartment volumes. These results prove that diabetes is detectable with deep learning from imaging data. Accordingly, 3-dimensional MRI images harbor patterns for a sufficiently good discrimination of patients with and without diabetes. Of note, the images were normalized for body length to target a classification based on fat distribution rather than body height.

In an empirical approach to look into the black box of machine learning, we applied human expert rating of heatmaps representing regions important for classification and regression. The interpretation of these heatmaps suggests that deep lower abdominal fat was most critical for the detection of diabetes by the machine (89% of diabetes heatmaps contained these areas). Furthermore, we also detected diabetes-related signals in the upper legs (66%), the arms (51%), and the neck region (51%).

Visceral fat, in contrast to subcutaneous fat, has been previously identified as an important predictor of insulin resistance, the failure to respond to lifestyle intervention and the future manifestation of diabetes (18, 19). Interestingly and somewhat unexpectedly, structures in the lower rather than upper abdomen turned out to be the most important topographic areas in our analyses. These results suggest that not all visceral adipocytes have the same impact on metabolism and point toward a heterogeneity with metabolically unfavorable fat enriched in the lower part. Highlights in the neck region could be linked to insulin resistance. Indeed, interscapular fat had been shown as an important independent marker of insulin resistance (20). Its importance in diabetes pathology is corroborated by our current hypothesis-free approach. The arms and upper leg regions are unforeseen hot spots, because they mostly comprise subcutaneous, metabolically inert fat depots. For estimation of BMI, arms and upper legs were the leading anatomic regions and might therefore predominantly represent general obesity. Of note, the upper leg region was also leading in the regression for insulin sensitivity (featured in 70% of heatmaps). Insulin resistance is the major body fat–derived metabolic factor in the pathogenesis of diabetes. However, insulin resistance is by itself not sufficient to cause diabetes (21). Diabetes only manifests if there is an additional disruption of pancreatic insulin secretion. Accordingly, we see a clear dissociation of the diabetes- and insulin resistance–related regions in our heatmaps. Unexpectedly, the deep lower abdomen differentiated diabetes from solitary insulin resistance. As the pancreas is not located in this area, our results suggest that additional biological signals that originate from the lower abdomen and target pancreatic islets could impair insulin release. The pancreas probably did not emerge directly in our machine-learning approach, as diabetes-related changes only occur in the islets that represent a minute proportion of the entire organ and can therefore hardly be detected by imaging. Another organ with known important contribution to diabetes, the liver, could correspond to highlighted areas in the deep right upper abdomen, appearing in 64% of diabetes with IFG+IGT classifiers. Accordingly, there was considerably less highlighting in the left upper abdomen, i.e., outside of the liver (13%). We have previously shown that a disruptive organ crosstalk among fat, liver, and pancreatic β cells could contribute to a deterioration of insulin secretion (13). Our findings about diabetes-related features of whole-body MRI stress the multiorgan nature of diabetes pathology.

The cluster analysis of the embeddings generated by machine learning from MRI scans shows a clear discrimination of 4 groups. This is not just a sole clustering of random image information but has biological meaning, as the clusters delineate different demographic and metabolic entities. As one of the identified subphenotypes was also associated with future diabetes and microalbuminuria, the most important early marker of diabetic kidney disease, the information content of the MRI images is also highly relevant for prediction of glycemic deterioration and a diabetes complication.

The results of sensitivity analyses using images restricted to the abdominal region suggest that future investigations could mainly focus on abdominal MR imaging. Using state-of-the art MR imaging techniques, higher resolution and faster acquisition times could be yielded, which might contribute to a better understanding of abdominal anatomy to diabetes pathology.

Our work has some limitations. Although different scanners were used to produce our data, this was a single-center study without external replication. Despite splitting our data into training, test, and replication sets, how our classifier will perform on data from different centers still needs to be tested. Furthermore, some of the data were repeated measurements in the same person, which we were unable to explicitly address in the machine-learning procedure. However, labels were updated concurrently (from oral glucose tolerance tests [OGTTs] performed at the time of each MRI scan), linking the respective metabolic status to anatomic patterns, and sensitivity analyses using a subset of the data without repeated measurements show comparable results for some labels. Capturing the intuition behind machine learning is still challenging, and there is no generally accepted method for this. To our knowledge, this is the first work to utilize 3-dimensional MRI whole-body scans for the analysis of diabetes and related features as well as to investigate a combination of heatmaps and their assignment to anatomic hot spots by human experts.

In summary, our work provides evidence that machine learning can classify diabetes from whole-body MRI. Diabetes, but not insulin sensitivity, was particularly associated with the features of the deep lower abdomen. This points toward considerable heterogeneity in the metabolic role of fat located in different parts of the visceral adipose tissue that has not been described so far. Further research is warranted on underlying molecular pathways that could represent important novel pathomechanisms in diabetes development.

## Methods

### Study population and MRI

MRI was performed on individuals who participated in metabolic screenings within the framework of multiple studies performed at the Department of Medicine IV, University Hospital Tübingen. In most of these studies, participants were generally healthy, without known type 2 diabetes but with an increased risk for the disease. This was defined as either family history of type 2 diabetes, BMI of greater than 27 kg/m2, or known prediabetes. The participants came fasted to the study facility and underwent whole-body MRI in the early morning, which was followed by a health examination, assessment of medical history, and an OGTT. OGTT does not only allow assessment of insulin sensitivity and glucose tolerance, but it is also the gold-standard detection of diabetes. Follow-up data for diabetes incidence and assessment of complications such as microalbuminuria was available for a subset of the subjects.

During the MRI, subjects were lying in a prone position with extended arms, and images were recorded from fingers to toes. A T1-weighted fast spin–echo technique with a slice thickness and an interslice gap of 10 mm was applied, allowing discrimination of adipose and lean tissue due to inherent different longitudinal relaxation times T1. The patient table was shifted by 10 cm after each measurement (12 seconds each). Total acquisition time, including 1 rearrangement, was 20–25 minutes (15).

### Data acquisition

Within the whole-body MRI scanning procedure, 90–120 parallel transverse slices were generated per participant, depending on body height. We quantified total adipose tissue volume, visceral adipose tissue volume, and upper extremities adipose tissue volume from these images for the benchmark models, using methods described previously (22). MR voxel arrays were provided in the Digital Imaging and Communications in Medicine file format. The original data set consisted of 2555 whole-body scans of 1080 participants, as some had been scanned multiple times. The number of MRI scans involved at different steps of the analysis is shown in Supplemental Figure 1. We used 8 ground truth labels. Four of these were binary labels; 1 was for sex, and the remaining 3 were for different diabetes definitions, including diabetes (Dα), prediabetes (Dβ), and a third definition, Dγ, that denoted diabetes cases extended with participants having concomitant IFG and IGT. In addition to the 4 binary labels, we used age (years), BMI (kg/m²), insulin sensitivity (determined with the Matsuda index; ref. 23), and glycated hemoglobin (HbA1c) (%) as target labels for the network. An overview of the characteristics of participants and the labels is provided in Supplemental Table 1. Laboratory measurements were performed as previously described (24). The diagnosis of diabetes was established by one of the following: fasting glucose >7.0 mmol/l, postchallenge glucose ≥11.1 mmol/l or a glycated hemoglobin ≥48 mmol/mol. Microalbuminuria was established by a uACR ≥30 mg/g creatinine.

### Data preprocessing

#### Shape normalization.

As mentioned, MRI scans were acquired by generating image slices along the body’s horizontal plane. Unlike the slice dimension, the number of slices varied according to body height. The most frequent number of slices was 95. All scans with different heights were linearly interpolated along the vertical body axis to produce volumes with normalized dimensions of 95 × 150 × 250 voxels. The voxel grid resolution of the 2 horizontal axes (considering a standing person) was considerably higher than the resolution along the body height axis, with negligible differences in coronal (*z* axis) scaling due to the aforementioned interpolation. We did not correct the lower resolution on the axis corresponding to the body height with further interpolations. However, we downsampled the standardized voxel grids for computational efficiency to their final dimension of 85 × 110 × 135 voxels.

#### Voxel value normalization.

Voxels that did not belong to the body (e.g., caused by motion artifacts inherent to MRI) were identified using value distributions and set to 0. We standardized body voxel values to have a mean of 0 and a SD of 1 and truncated and subsequently shifted the distribution to strictly positive values to keep the distinction from the surrounding air. We transformed all scan samples equally.

### Labels

A total of 8 outcome label variables were used as described. Samples with missing labels were excluded, continuous variables were normalized to a range between 0 and 1. Outliers were removed using the isolation forest algorithm and fitted on a subset of the medical features, namely insulin sensitivity, BMI, HbA1c, as well as total adipose tissue estimate (25).

### Data partitioning

In order to assess the generalization capabilities of our models, we applied a stratified random split to separate the entire data set into training (70%), validation (15%), and test (15% of all data) folds. The folds were stratified by BMI, insulin sensitivity, and diabetes. Our stratification algorithm required that each multivariate stratum contains more than 1 sample (Supplemental Figure 1).

### Augmentation

To increase model robustness, the input volumes were augmented in several ways. Additional 0 padding was added to each dimension, increasing the size of the 3-dimensional image array. To augment the training samples, the degree of padding was dynamically adjusted at random during training. Furthermore, a series of rotations were randomly performed on each input array as well as addition of Gaussian noise to all body voxels (Supplemental Figure 2). For testing and validation, the body volumes were centered, and no rotation or noise was applied. We also performed sensitivity analyses using additional random zooming of the images during the training. Furthermore, we tested the training on restricted images cropped to the torso and abdominal area (Supplemental Figure 3, A and B).

### Model architecture

#### Densely connected convolutional layers.

We built the network in accordance to the DenseNet architecture (26). The 3-dimensional input volumes were fed to the input layer in batches consisting of 8 samples each. The initial layer consisted of a fully connected convolutional layer with a kernel size of 5 and 8 convolutional filters. The initial convolutional layer was followed by a batch normalization layer (27, 28). The dimensions of the intermediary feature maps were subsequently downsampled using a pooling layer to improve computation efficiency. The output was fed to the first dense block. Alternating dense blocks and transition layers were sequentially added to process the input.

Following the final transition layer, the activation maps were flattened to a 1-dimensional array and passed to 3 sequential densely connected layers with dropout. The output of the dense layers had the dimension 1 × 128 units and was referred to as the embedding layer, embodying low-dimensional representations of MRI voxels as “learned” by the neural network. The embedding layer was used for the prediction of the desired target labels and for unsupervised clustering analysis. For the prediction of the output nodes, subsequent dense layers were added to the embedding layer. A schematic of the entire model is provided in Supplemental Figure 2.

#### Gradient heatmaps.

Predictions for the various target variables were directly represented by the output nodes. Differentiating these with respect to previous convolutional layers yields pixel-wise gradients. The chosen approach for heatmap generation was gradients × input (27); hence, we computed the gradients of the individual outputs with respect to the image input. Differentiating resulted in target-specific gradients of the same dimension as the input scans. Gradient heat maps were assigned to anatomical regions by 3 human medical experts (experienced physicians working in hospitals) who had experience in the evaluation and interpretation of medical imaging. All expert raters were blinded to the subject characteristics as well as to the trait they rated. Results were averaged for the 3 raters.

#### Hyperparameters.

We used a growth factor kGrowth = 18. The initial convolution layer, prior to the first dense block had a kernel size of 5 × 5 × 5 voxels, and it generated 4 activation maps. We chose to evaluate the model with 3 dense blocks and 3 subsequent fully connected layers, downsampling the flattened representation to 512, 256, and 128, respectively. With the sole exception of the final regression output layers, all activation functions throughout the network were exponential linear units (28). We chose the initialization proposed by He et al. (29) for all kernel weights. Adam (30) was used as optimizer with an initial learning rate of 10-4. All other hyperparameters of the optimizer were kept at their Tensorflow (31) implementation defaults. The learning rate is adapted during training through a Tensorflow variable and has a cyclic, exponentially decay.

#### Training.

Network training was performed on a Nvidia Tesla V100-PCIE 32GB GPU, using the CUDA framework (32). The network was trained for a maximum of 250 epochs with a batch size of 8, due to the considerable memory requirements of our 3-dimensional voxel grids. The network converged after approximately 2–3 days, depending on network depth, i.e., number of trainable parameters as well as batch size and other hyperparameters.

#### Model selection.

We frequently evaluated the network’s performance and selected the model according to the highest diabetes (Dα) AUROC score on the validation set.

**Figure 4:**
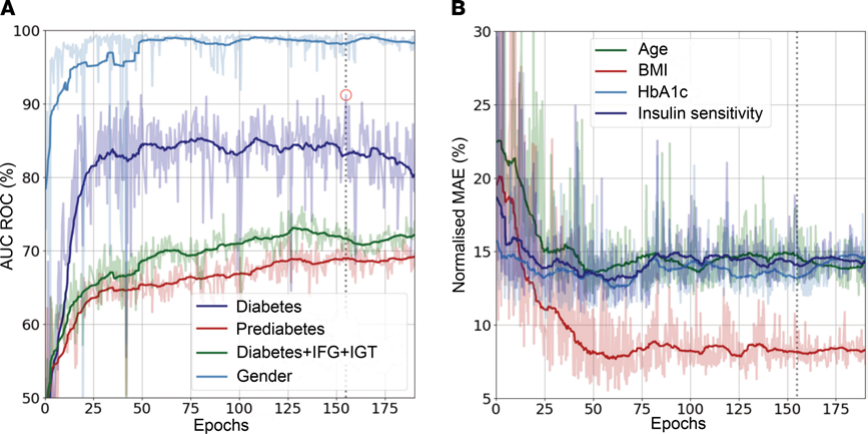
Training metrics and model selection. Performance of models in subsequent computation runs for classifications (area under the receiver operating characteristic (ROC) curves (**A**) and regressions (normalized mean absolute error) (**B**). The circled point in **A** indicates the highest achieved ROC for diabetes in the validation set.

We first summarized the training progress, using the AUROC and MAE metric on the validation set over all trained epochs (Figure 4). Metrics were generally computed on the test set with the exception of diabetes (Dα) and prediabetes (Dβ). Due to the critically low number of positives for these labels, we chose to concatenate the data sets for testing and validation to compute the classification performance metrics. To assess the performance of our approach, benchmark models were computed using linear regression and k-nearest neighbor classifier, random forest models, and support vector machines for comparison. Body fat compartment volumes that have been segmented from MR images (total, visceral, and upper limb adipose tissue) were used as model inputs.

#### Postprocessing.

The output of the model consisted of a set of predictions for each sample in addition to the respective gradient maps as well as its embedding space representation.

We used gradient maps to compute target specific heat maps, using the gradients×input method, proposed by Shrikumar et al. (27). The individual output nodes were differentiated with respect to the input to produce 3-dimensional feature-specific gradient maps. For visualization, the gradient maps were postprocessed using Gaussian filters in addition to contrast enhancements and averaging to 2 dimensions. Furthermore, for the classification nodes, only the output node corresponding to the correct label was considered. In other words, for a patient with label female, only the gradient that increased the female probability prediction was used for visualization. All positive gradients were considered for the regression tasks.

### Data availability

All requests for data and materials are promptly reviewed by the Data Access Steering Committee of the Institute of Diabetes and Metabolic Research, Tübingen, Germany, to verify if the request is subject to any intellectual property or confidentiality obligations. Individual level data may be subject to confidentiality. Any data and materials that can be shared will be released via a material transfer agreement.

### Statistics

Cluster analysis was performed on the embeddings using partitioning around medoids with Gower’s distances. The optimal number of clusters was selected using average Silhouette widths. To investigate the robustness of the clusters, we performed a bootstrap validation showing a Jaccard similarity index of 0.73 over all clusters. A subset of the participants was tested during follow-up visits for incident diabetes (*n* = 586) and microalbuminuria (*n* = 550). Comparison of risks was performed using Kaplan-Meier diagrams and log-rank tests. Further analyses with adjustments for potential confounders were performed with proportional hazards models. Proportional hazards assumptions were tested by visualizing Schoenfeld residuals.

### Study approval

The studies were carried out with written informed consent from all subjects in accordance with the Declaration of Helsinki. All protocols were approved by the ethics committee of the University of Tübingen.

## Author contributions

JM and RW contributed to data acquisition, analyzed the data, and wrote the manuscript. BD, VA, and SB analyzed the data and wrote the manuscript. JD and SR contributed to data acquisition and the interpretation of data and edited the manuscript. MH, HUH, and AF contributed to the concept of the work and edited the manuscript. PS, ALB, FS, NS, HP, and BS contributed to the interpretation of data and edited the manuscript. All authors have reviewed the manuscript. BD constructed the first machine-learning models and is therefore listed first among the co–first authors.

## Supplementary Material

Supplemental data

## Figures and Tables

**Table 1:**
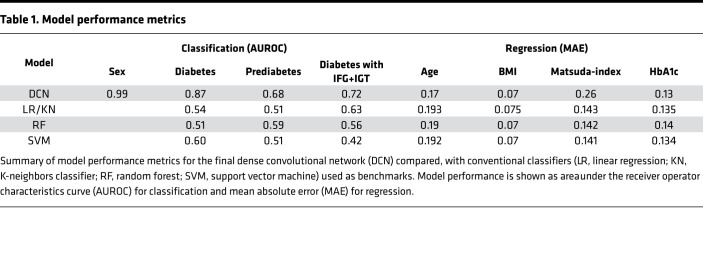
Model performance metrics

**Table 2:**
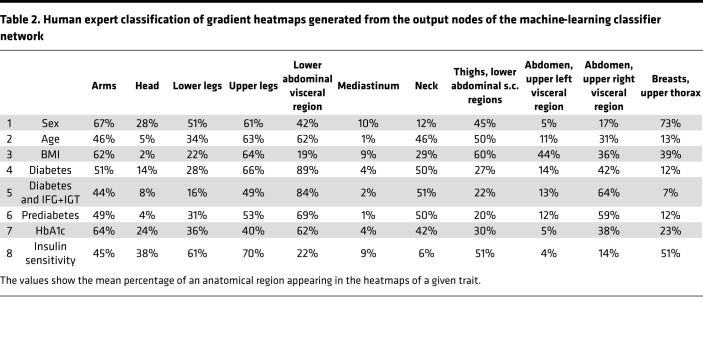
Human expert classification of gradient heatmaps generated from the output nodes of the machine-learning classifier network

**Table 3:**
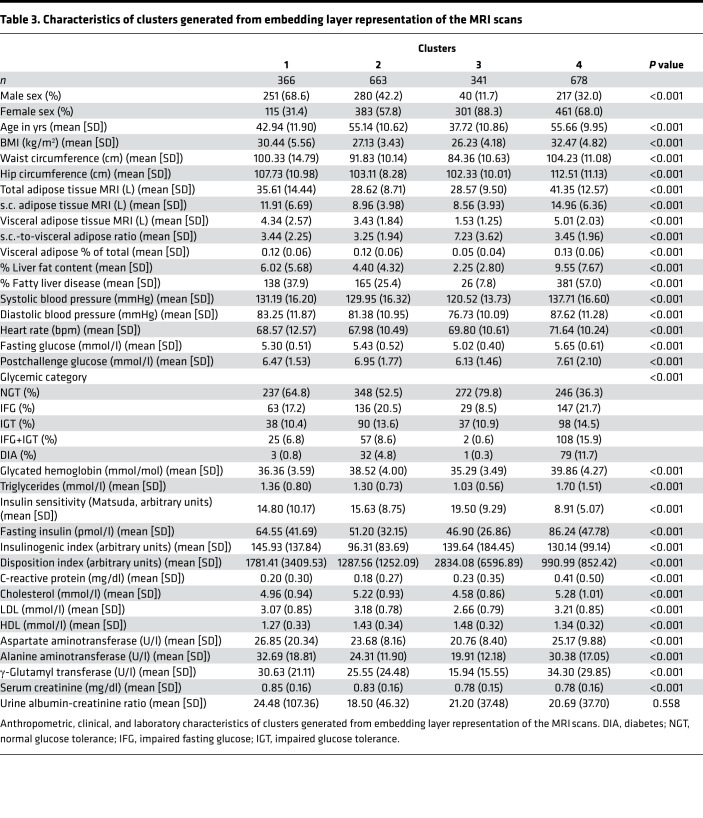
Characteristics of clusters generated from embedding layer representation of the MRI scans

## Version 1 09/30/2021

In-Press Preview

## Version 2 11/08/2021

Electronic publication
