# Supplementary material for: Detection of diabetes from whole-body MRI using deep learning
Source: JCI Insight. 2021 Nov 8;6(21):e146999. doi: 10.1172/jci.insight.146999 (PMC8663560; doi:10.1172/jci.insight.146999)
Supplement: Supplemental data [file jciinsight-6-146999-s127.pdf]

Supplementary Data to the Manuscript

Diabetes detection from whole-body magnetic resonance imaging using deep learning

by

Benedikt Dietz[1*], Jürgen Machann, PhD[2,3,4*], Vaibhav Agrawal[5,6], Martin Heni, MD[3,4,7,8] Patrick Schwab[9], Julia Dienes, MD[10], Steffen Reichert, MD[3,4,8], Andreas L. Birkenfeld, MD[3,4,8], Hans-Ulrich Häring, MD[3,4], Fritz Schick, MD, PhD[2,3,4], Norbert Stefan, MD[3,4,8], Andreas Fritsche, MD[3,4,8], Hubert Preissl, PhD[3,4,8], Bernhard Schölkopf, PhD[6], Stefan Bauer, PhD[,611**] and Robert Wagner, MD[3,4,8**]

[1] Department of Computer Science, ETH Zürich, Universitätstrasse 6, 8092 Zürich, Switzerland

[2.] Department of Radiology, Section on Experimental Radiology, Eberhard-Karls University Tübingen, Hoppe-Seyler-Str. 3, 72076 Tübingen, Germany

[3] Institute for Diabetes Research and Metabolic Diseases of the Helmholtz Center Munich at the University of Tübingen, Otfried-Müller-Str. 10, 72076 Tübingen, Germany

[4.] German Center for Diabetes Research (DZD e.V.), Ingolstädter Landstraße 1, 85764 Neuherberg, Germany

[5] Werner Siemens Imaging Center, Röntgenweg 13, 72076 Tübingen, Germany

[6] Max Planck Institute for Intelligent Systems, Department of Empirical Inference, Max-Planck-Ring 4, 72076 Tübingen, Germany

[7] Department for Diagnostic Laboratory Medicine, Institute for Clinical Chemistry and Pathobiochemistry, University Hospital Tübingen, Otfried-Müller-Str. 10, 72076 Tübingen, Germany

[8] Department of Internal Medicine, Division of Diabetology, Endocrinology and Nephrology, Eberhard-Karls University Tübingen, Otfried-Müller-Str. 10, 72076 Tübingen,Germany

[9] Institute of Robotics and Intelligent Systems, ETH Zürich, Tannenstrasse 3, 8092 Zürich, Switzerland

[10] Department of Gynecology and Obstetrics, University Hospital Tübingen, Calwerstraße 7, 72076 Tübingen, Germany

[11] Department of Intelligent Systems, KTH Stockholm, Malvinas Väg 10, 114 28 Stockholm, Sweden

Contents

Diabetes detection from whole-body magnetic resonance imaging using deep learning 1

Contents 2

Supplementary Figures 3

Supplementary Figure 1 3

Supplementary Figure 2 3

Supplementary Figure 3 5

Supplementary Tables 6

Supplementary Table 1 6

Supplementary Table 2 8

Supplementary Figures

Supplementary Figure 1

Study flow chart on the pre-processing of available images

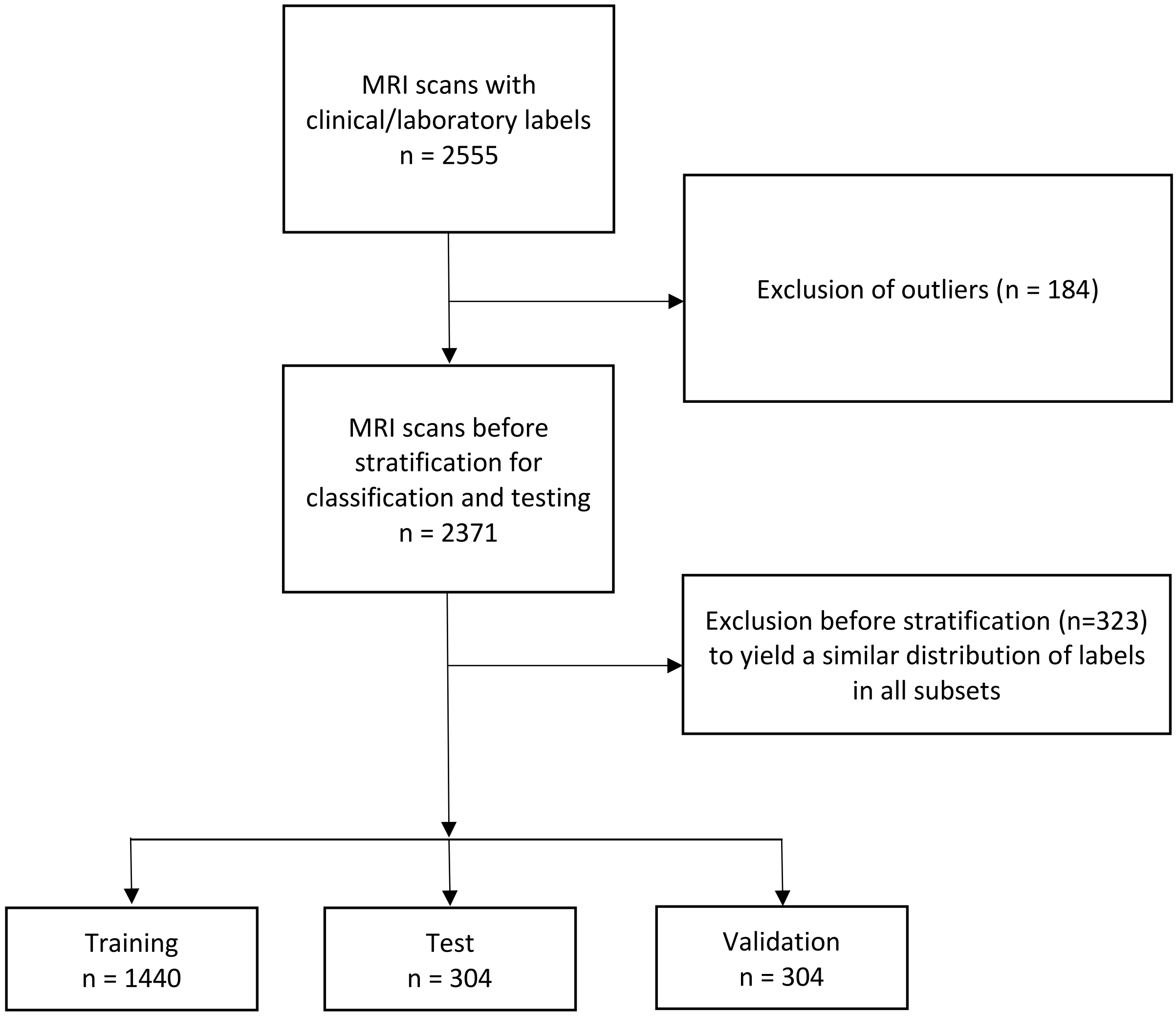

Supplementary Figure 2

Schematic of Model Architecture.

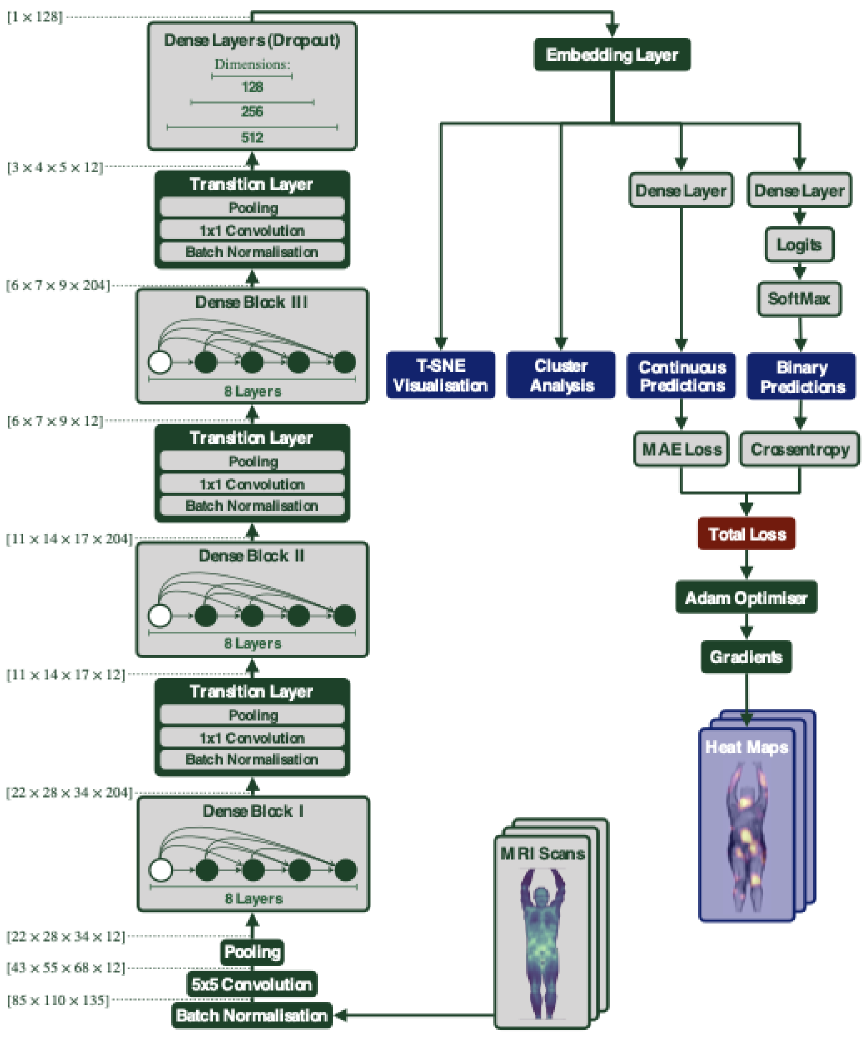

The input to the network consisted of mini-batches of three-dimensional MRI scans. The input was passed to a batch normalisation layer as well as an initial convolution and pooling operation. Afterwards, three dense blocks were built in series with transition layers following each dense block. Finally, we used a series of densely connected layers with dropout to map the output of the final transition layer to the embedding layer of dimension 1x128 (Dimensions between network layers were denoted on the left). The embedding layer was used as input for post-processing and predictions. We optimized the sum of all prediction losses to optimize the network and used the trained model to generate gradient maps.

Supplementary Figure 3

Restricted anatomic regions selected for alternative training approaches (A. Torso, B. Abdominal region). For results, see Supplementary Table 2.

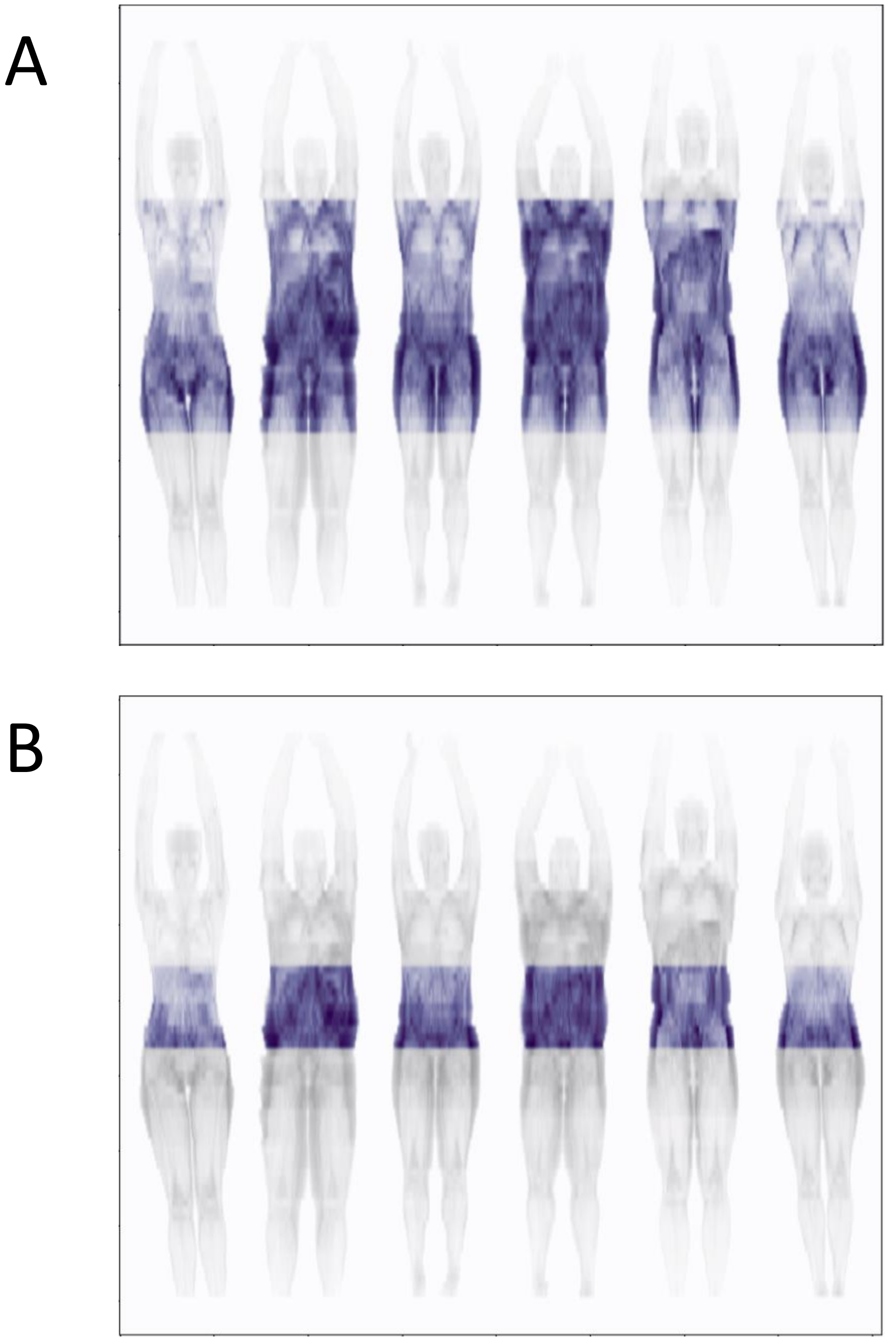

Supplementary Tables

Supplementary Table 1

Demographic, clinical and laboratory data corresponding to the MR images used for training, test and validation

	Overall
Number of scans	2048
Number of study participants	790
subjects according to number of measurements (%)	
1 measurement	265 (33·6)
2 measurements	201 (25·4)
3-4 measurements	218 (27·6)
>4 measurements	106 (13·4)
elapsed days since previous measurement (mean (SD))	419·07 (732·78)
sex = male (%)	788 (38·5)
age (mean (SD))	50·23 (12·93)
BMI (kg/m2) (mean (SD))	29·34 (5·15)
waist circumference (cm) (mean (SD))	96·21 (13·62)
hip circumference (cm) (mean (SD))	106·92 (10·95)
systolic blood pressure (mean (SD))	131·17 (17·00)
diastolic blood pressure (mean (SD))	83·01 (11·72)
heart rate (mean (SD))	69·60 (10·93)
fasting glucose (mmol/l) (mean (SD))	5·41 (0·58)
post-challenge glucose (mmol/l) (mean (SD))	6·95 (1·88)
glycemic category (%)	
NGT	1103 (53·9)
IFG	375 (18·3)
IGT	263 (12·8)
IFG+IGT	192 (9·4)
DIA	115 (5·6)
glycated hemoglobin (mmol/mol) (mean (SD))	38·04 (4·30)
triglycerides (mmol/l) (mean (SD))	1·40 (1·07)

	Overall
insulin sensitivity (Matsuda) (mean (SD))	13·90 (8·98)
fasting insulin (pmol/l) (mean (SD))	64·47 (42·24)
insulinogenic index (mean (SD))	123·59 (122·24)
disposition index (mean (SD))	1535·14 (3233·94)
cholesterol (mmol/l) (mean (SD))	5·09 (0·98)
LDL (mmol/l) (mean (SD))	3·08 (0·84)
HDL (mmol/l) (mean (SD))	1·38 (0·34)
aspartate-aminotransferase (mean (SD))	24·25 (11·94)
alanine-aminotransferase (mean (SD))	27·09 (15·81)
gamma-glutamyl transferase (mean (SD))	27·75 (25·47)
serum creatinine (mg/dl) (mean (SD))	0·81 (0·16)

Supplementary Table 2

Diagnostic precision of different model setups showing AUC-ROC results for four labels. Zooming was performed by applying random zooming on the image.

	Gender	Prediabetes	Diabetes	Diabetes with IFG+IGT
**All data with repeated measurements**				
Whole body, default augmentation	0.99	0.69	0.87	0.72
Zooming	0.99	0.62	0.87	0.71
Torso only*	0.99	0.60	0.67	0.74
Torso only* with zooming	0.99	0.63	0.77	0.73
Abdomen only**	0.99	0.61	0.83	0.71
Abdomen only** with zooming	0.99	0.63	0.71	0.70
**One measurement per participant**				
Whole body, default augmentation	0.97	0.68	0.79	0.71

Restricted image analyses were performed with *torso only (neck to upper thigh region excluding the extremities, see Supplementary Figure 3.A) and **abdomen only (diaphragm to hip, see Supplementary Figure 3.B).
